# Investigating the Dynamic Mechanical Properties and Strengthening Mechanisms of Ti-6Al-4V Alloy by Using the Ultrasonic Surface Rolling Process

**DOI:** 10.3390/ma17061382

**Published:** 2024-03-18

**Authors:** Xuming Zha, Zhi Yuan, Hao Qin, Linqing Xi, Yunwu Guo, Zhilong Xu, Xing Dai, Feng Jiang

**Affiliations:** 1 College of Marine Equipment and Mechanical Engineering, Jimei University, Xiamen 361021, China; 202111802008@jmu.edu.cn (Z.Y.); 202211802018@jmu.edu.cn (H.Q.); xlq15552145569@163.com (L.X.); 202311802011@jmu.edu.cn (Y.G.); zhilong.xu@163.com (Z.X.); 2 State Key Laboratory of Intelligent Manufacturing Equipment and Technology, Huazhong University of Science and Technology, Wuhan 430074, China; 3 HUST-Wuxi Research Institute, Wuxi 214174, China; daixing_hust@163.com; 4 Institute of Manufacturing Engineering, Huaqiao University, Xiamen 361021, China

**Keywords:** ultrasonic surface rolling process, residual compressive stress, fatigue performance, dynamic mechanical properties, strain rate

## Abstract

The demand for titanium alloy has been increasing in various industries, including aerospace, marine, and biomedical fields, as they fulfilled the need for lightweight, high-strength, and corrosion-resistant material for modern manufacturing. However, titanium alloy has relatively low hardness, poor wear performance, and fatigue properties, which limits its popularization and application. These disadvantages could be efficiently overcome by surface strengthening technology, such as the ultrasonic surface rolling process (USRP). In this study, the true thermo-mechanical deformation behavior of Ti-6Al-4V was obtained by dynamic mechanical experiment using a Hopkinson pressure bar. Moreover, USRP was applied on the Ti-6Al-4V workpiece with different parameters of static forces to investigate the evolution in surface morphology, surface roughness, microstructure, hardness, residual stress, and fatigue performance. The strain rate and temperature during the USRP of Ti-6Al-4V under the corresponding conditions were about 3000 s^−1^ and 200 °C, respectively, which were derived from the numerical simulation. The correlation between the true thermo-mechanical behavior of Ti-6Al-4V alloy and the USRP parameters of the Ti-6Al-4V workpiece was established, which could provide a theoretical contribution to the optimization of the USRP parameters. After USRP, the cross-sectional hardness distribution of the workpiece was shown to initially rise, followed by a subsequent decrease, ultimately to matrix hardness. The cross-sectional residual compressive stress distribution of the workpiece showed a tendency to initially reduce, then increase, and finally decrease to zero. The fatigue performance of the workpiece was greatly enhanced after USRP due to the effect of grain refinement, work hardening, and beneficial residual compressive stress, thereby inhibiting the propagation of the fatigue crack. However, it could be noted that the excessive static force parameter of USRP could induce the decline in surface finish and compressive residual stress of the workpiece, which eliminated the beneficial effect of the USRP treatment. This indicated that the choice of the optimal USRP parameters was highly crucial. This work would be conducive to achieving high-efficiency and low-damage USRP machining, which could be used to effectively guide the development of high-end equipment manufacturing.

## 1. Introduction

Titanium alloys fulfill the need for lightweight, high-strength, and corrosion-resistant material for modern manufacturing. As a result, the demand for titanium alloy has been increasing in aerospace [1,2], marine [3,4], and biomedical fields [5,6]. During the service period of titanium alloy components under complex conditions, fatigue failure is one of the main reasons for inducing damage and fracture of the components, which could result in serious consequences [7]. Numerous studies have shown that surface strengthening methods have the potential to enhance the fatigue life of key components. The surface strengthening methods for practical engineering applications mainly consist of shot peening [8], laser shock peening [9], and ultrasonic surface rolling process [10]. Shot peening has been commonly applied in scientific research and manufacturing because of its cost-effectiveness and suitability for processing complex-shaped components. By forming a residual compressive stress layer and a gradient layer on the material surface after shot peening, the fatigue performance of components was significantly improved. However, due to the randomness of the shot peening processing with many shots projected on the material, the formation of the residual compressive stress layer could be uneven distribution, and the surface quality could deteriorate. Laser shock peening involves using a high-energy laser beam to act on an absorbing layer attached to the surface of the workpiece material. This process generates a high-temperature, high-pressure plasma that causes localized expansion and explosion. The high-energy shock wave formed by localized expansion and explosion could operate on the workpiece surface under the constraint of a confinement layer. However, the pre-processing stage of the practical laser shock peening process is relatively complicated. Furthermore, the overall price of the laser shock strengthening equipment is very high, which leads to higher manufacturing costs. In contrast to the two strengthening techniques mentioned above, the ultrasonic surface rolling process (USRP) was characterized as the coupled influence of static force and dynamic ultrasonic impact force applied on the workpiece. The plastic deformation of the workpiece could be facilitated by an ultra-high-frequency (≥20 kHz) vibratory impact of the rolling ball, and the strain rate could achieve 10^2^ s^−1^ to 10^3^ s^−1^ during this condition [11]. Simultaneously, the homogeneous surface deformation could be attributed to the rotational movement of the processed material [12]. USRP could significantly decrease the surface roughness and raise the geometrical precision of the machined surface, thus greatly enhancing the machining quality. Additionally, USRP could promote the development of a gradient structure with the compressive residual stress field and work-hardening layer. Currently, USRP has effectively been utilized to enhance the fatigue properties of a variety of aerospace metals [13,14,15], especially titanium alloys. 

Based on the strain hardening theory, when the material surface was subjected to true stress that exceeded its yield strength, the micro-plastic deformation behavior could occur during USRP treatment. This could induce an increase in the internal dislocation density and a decrease in the grain size of the material subsurface so the mechanical properties of the workpiece material could be enhanced. It is of great significance to establish the correlation between the true thermo-mechanical behavior of the material and the ultrasonic surface rolling process of the workpiece. In recent years, some researchers have investigated the mutual influence relationship between the quasi-static mechanical properties and the ultrasonic impact strengthening process. Liu et al. [16] simulated a single-point ultrasonic impact-strengthening process. The results demonstrated a positive correlation between the number of impacts, the rise in residual stress, and the corresponding plastic strain. The mechanical characteristics of the materials were measured under cyclic tensile-compression experiments at low strain rates. However, the strain rate of ultrasonic impact strengthening was approximately 10^2^/s–10^3^/s, so it could be hard to obtain the material response adequately under the high-velocity impact condition during USRP treatment. In addition, it was worth mentioning that the USRP was characterized as a conjunction of the static force and the dynamic ultrasonic impact force, causing a significant change in the transient velocity and acceleration of the rolling ball. As a result, plastic deformation with high strain rates was generated at the workpiece surface. The mechanical behavior of the material was significantly different between high strain rate deformation and quasi-static deformation conditions. Several scholars have developed material constitutive models to characterize the deformed behaviors of material based on experimental law. Liu et al. [17] obtained the conventional J-C model of 18CrNiMo7-6 alloy steel by mechanical experiments at different strain rates. It found that the conventional J-C constitutive model could not precisely forecast the flow stress under high strain rate loading conditions. The error of stress value between the prediction and the test grew gradually as the strain rate increased. Thus, a revised J-C model was established. The strain rate strengthening coefficient was modified in a non-linear adjustment, taking into account the interdependence between strain and the strain-rate coefficients. At a strain rate of 3500/s, the average absolute relative error between the true stress predicted by the revised J-C model and the test of the true stress was reduced from 6.9% to 3.2%. The revised J-C model could accurately assess the high strain rate and mechanical behavior of 18CrNiMo7-6 alloy steel during USRP. Teimouri et al. [18] established an analytical model of the ultrasonic surface burnishing process to investigate the consequences of various factors on the residual stress field. Two different constitutive models were chosen to validate the accuracy of this elastic–plastic analytical model, i.e., the J-C model, which was susceptible to strain rate, and the Chaboche hardening model, which was affected by cyclic loading. The results indicated that the J-C model showed more consistency in predicting the residual stress field, whereas the Chaboche hardening model underestimated the predictions. In addition, the forecast of the residual compressive stress field revealed that the depth and magnitude of compressive residual stress exhibited a positive correlation with the amplitude, static force, and ball diameter. In another work, Teimouri et al. [19] established the concept of an elastoplastic cavity expansion model of materials (ECM) based on the physical model of the AA6061 aluminum alloy. The residual stress field was further calculated by this model with the aim of discovering the correlation between the amplitude and the residual stress field. The results indicated that raising the vibration amplitude value led to a greater degree of residual compressive stress introduction. Furthermore, it was found that augmenting the magnitude of vibration led to an increase in the strain rate at which the material surface underwent plastic deformation. Zhou et al. [20] investigated the transient process by establishing a 3D FEM model of ultrasonic impact processing. It was noted that plastic deformation of the material did not occur with every single impact. This phenomenon only took place when the impact kinetic energy was above certain threshold levels. Increasing the velocity of the pin intensified the impact kinetic energy, resulting in significant plastic deformation of the workpiece. The maximum strain rate of a material undergoing plastic deformation during ultrasonic impact was 1300/s. Luan et al. [11] conducted a Hopkinson pressure bar test to investigate the dynamic mechanical characteristics of 45CrNiMoVA steel at elevated temperatures. By extracting eigenvalues on the stress–strain curve, the relevance between dynamic mechanical characteristics and strain rate could be constructed. The findings demonstrated that the flow stress of 45CrNiMoVA reduced substantially as the temperature increased. The corresponding yield strength, ultimate strength, and modulus of elasticity exhibited a decreasing tendency. This indicated that the material surface was more susceptible to plastic deformation during the USRP at high temperatures, which could result in a deeper plastic deformation layer. Wu et al. [21] established an analytical model and analyzed the relationship between static load and strain value during the ultrasonic impact. The static load could be determined from the maximum strain value between the yield strength and ultimate strength of the 20CrNiMo stress–strain curve. Chan et al. [22] studied the microstructure of 304 stainless steel after ultrasonic peening by TEM and analyzed the association with the strain rate. The findings indicated that the high strain rate of plastic deformation could be conducive to producing a comparatively higher density of dislocations and nano-twins in the surface layer. According to the above research, it could be implied that the strain rate during high-speed impact surface treatment is essential to optimizing the microstructure of the surface layer, thus optimizing the mechanical properties of the workpiece. The optimal USRP parameter range could be determined depending on the thermo-mechanical performance of the material. 

The service performance of the material after the USRP treatment was significantly improved. The current authors have reported that the strengthening mechanisms of material fatigue performance by the USRP treatment mainly included three reasons: (Ⅰ) USRP induced the residual compressive stress field at the workpiece surface, which could be helpful in inhibiting the beginning of fatigue crack and reducing the crack extension rate [23,24]; (Ⅱ) USRP caused grain refinement and hardness enhancement at the subsurface of the workpiece. The combined effect of the deep harden layer and grain refinement layer could effectively prevent fatigue crack propagation [25,26]; (Ⅲ) USRP further decreased the influence of stress concentration by reducing the surface roughness of the workpiece after the cutting process [27,28]. The reduction in stress concentration factor plays an essential function in increasing the fatigue properties of the workpiece. Zhao et al. [29] discovered that USRP could form nanostructured grain boundaries on the surface of TC11 alloy, restricting the movement of dislocations and enhancing the difficulty of the initiation of the dislocation source. As a result, the resistance to deformation of the TC11 alloy has improved. The fatigue life was increased to 19.3% under the number of cycles with 5×10^6^ in the fatigue testing. Dekhtyar et al. [30] showed that the fatigue life of powder metallurgy Ti-6Al-4V alloy was extended approximately by a factor of 100 after USRP at fatigue cyclic stress amplitudes of 300–400 MPa. The increased fatigue strength and service life after the USRP could be attributed to modifications in the reduced surface roughness, the microstructure, and the residual stress state at the surface. In addition, the USRP could induce significant pore closure in the deformation layer about 200–250 μm thick, which had a beneficial effect on lifetime extension. Kattoura et al. [31] studied the fatigue performance of the ATI718 alloy at elevated temperatures after ultrasonic nanocrystal surface modification (UNSM). The application of UNSM resulted in significant surface plastic deformation, causing the production of crystallites and twins at the nanoscale in the near-surface areas. This was accompanied by an increase in the hardness of the workpiece and the existence of substantial compressive residual stresses. The residual stresses in the material could be released at high temperatures, but these microstructures could remain stable at high temperatures. The yield strength and the fatigue strength were enhanced by 11% and 8%, respectively, in the fatigue testing at 650 °C because of the stable microstructure and retained residual stresses resulting from the UNSM treatment. As a result, UNSM could increase the fatigue resistance of the ATI718 alloy at high temperatures. This improvement might be caused by the synergistic influence of residual compressive stresses and temperature-stabilized work hardening.

The novelty of this work is to establish the correlation between the true thermo-mechanical behavior of the material and the USRP parameters of the workpiece. In this study, the Hopkinson pressure bar was utilized to investigate the dynamic deformation behavior of the Ti-6Al-4V alloy under thermo-mechanical processing. Additionally, the numerical simulation examined the evolution of the deformation strain rate and contact temperature between the rolling ball and Ti-6Al-4V material during the USRP. Combined with the results of the dynamic true stress–strain testing experiment, it could contribute to obtaining the relatively accurate deformation regularity of Ti-6Al-4V and investigating the strengthening mechanisms under the actual working conditions. Further, changes in the surface roughness, microstructure, hardness, and residual stress after USRP and rotational bending fatigue testing were analyzed utilizing scanning electron microscopy (SEM), laser scanning confocal microscope (LSM), X-ray diffraction (XRD), and so on. Fracture modes and mechanisms were also characterized by SEM to investigate the influence of USRP on fatigue life improvement.

## 2. Experimental Scheme

### 2.1. Material of Workpiece

**Figure 1:**
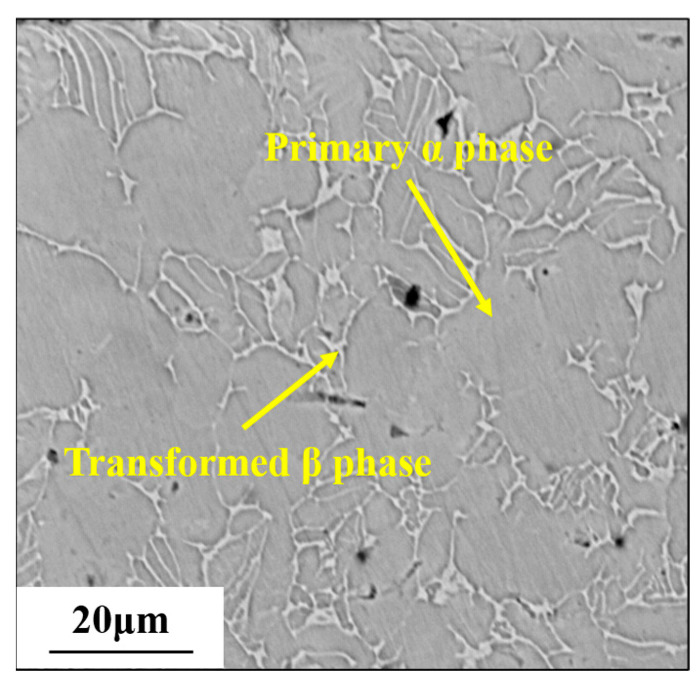
SEM image of original microstructure of Ti-6Al-4V.

**Figure 2:**
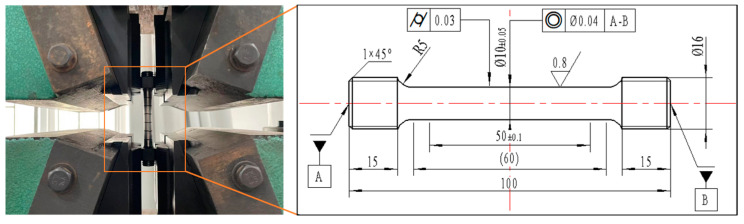
The shape of tensile test specimen.

**Figure 3:**
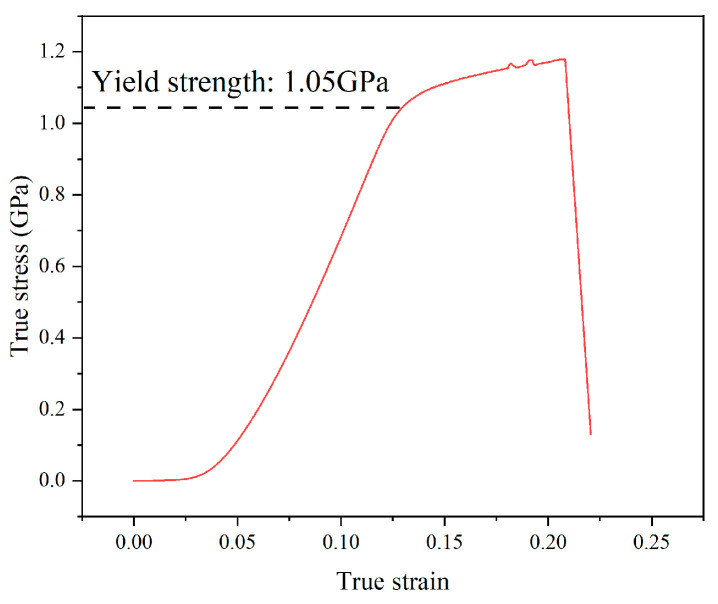
Quasi-static tensile test results of Ti-6Al-4V alloy.

The commercial Ti-6Al-4V alloy with a diameter of 20 mm suffered the vacuum annealing process for 1 h at a temperature of 780 °C. Subsequently, it was allowed to cool down to ambient temperature in the natural environment. Figure 1 illustrates the original microstructure of Ti-6Al-4V, which consists of equiaxed primary α phase and transformed β phase. The chemical composition of the Ti-6Al-4V workpiece is listed in Table 1. In order to obtain the yield strength of the original workpiece, quasi-static tensile tests were carried out at room temperature (25 °C) on the tensile test machine (WDW-100, Changchun Kexin Test Instrument Co., Changchun, China). Figure 2 shows the shape of the tensile test specimen. The specimen was fixed in the tensile test machine. An axial tensile load was applied on both ends of the tensile specimen at a strain rate of 0.01 s^−1^. The yield strength of the original workpiece was 1.05 GPa, which could be obtained from Figure 3.

### 2.2. Dynamic Mechanical Properties Testing Method

**Figure 4:**
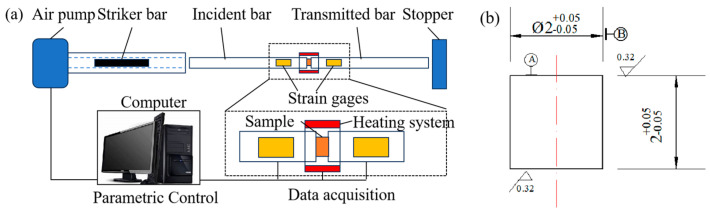
Structural schematic of Hopkinson pressure bar experiment platform (**a**) and the size of the sample (**b**).

The USRP treatment was characterized by the periodic impact loads applied to the workpiece surface, leading to surface deformation at a high strain rate. Moreover, it was worth mentioning that the Ti-6Al-4V alloy was particularly sensitive to strain rates. In this research, the dynamic mechanical characteristics of the workpiece under various strain rates and temperatures were measured by the Hopkinson pressure bar. Figure 4 displays the structural schematic of the Hopkinson pressure bar experiment platform. The sample was clamped between the incident bar and the transmitted bar. The size of the cylinder Ti-6Al-4V alloy sample is Ø2 × 2 mm, as shown in Figure 4b. Due to the friction effect between the rolling ball and the specimen, an accumulation of heat energy was generated on the surface of the specimen, which characterized USRP as a thermo-mechanical coupling process. Thus, the high strain rate impact experiments at different temperatures of Ti-6Al-4V alloy were employed. The Hopkinson pressure bar was used to perform the dynamic mechanical tests at room temperature and elevated temperatures, respectively. The sample was placed in the heating system, as shown in Figure 4a. The experimental temperature was set as 25 °C (room temperature), 200 °C, and 400 °C, respectively. The heating temperature was measured by a platinum–rhodium thermocouple. The distance between the thermocouple and the sample surface was approximately 1 mm. Prior to starting the dynamic mechanical test, the sample was heated and held at the testing temperature for 10 min. Table 2 shows the parameters of the dynamic mechanical properties test.

### 2.3. Ultrasonic Surface Rolling Processing

**Figure 5:**
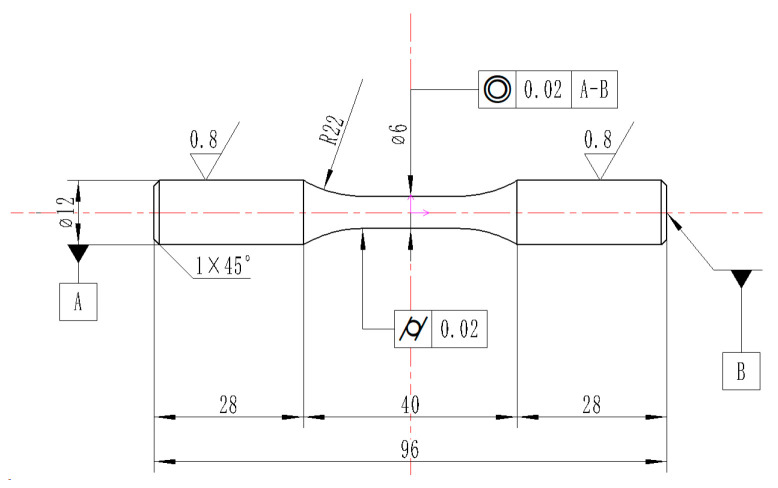
The dimensions of the fatigue sample.

**Figure 6:**
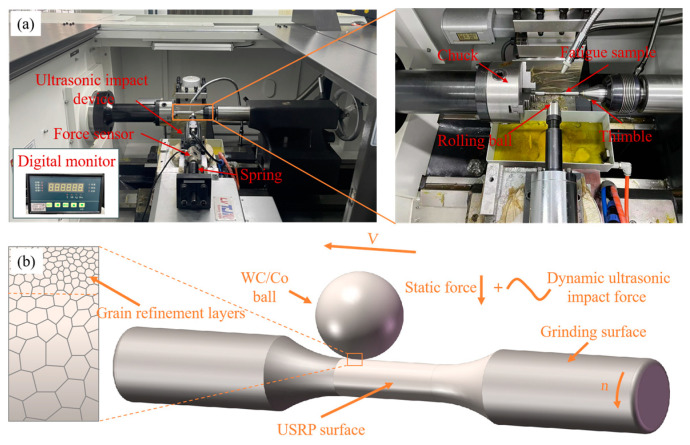
(**a**) USRP experimental setup and (**b**) Schematic of USRP process.

**Figure 7:**
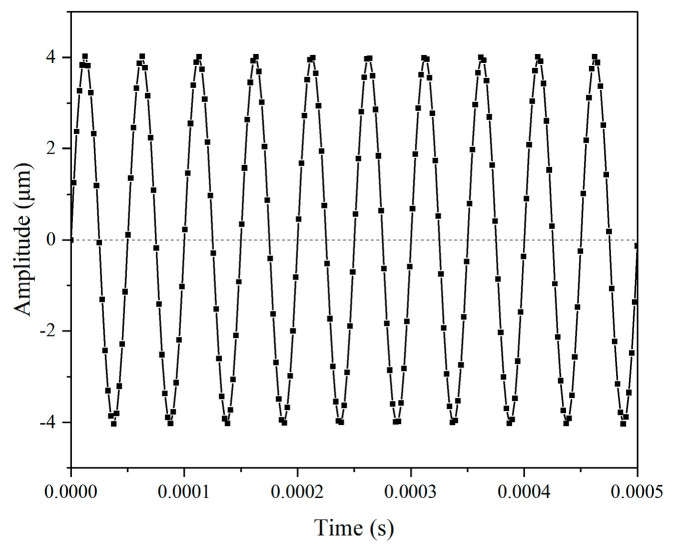
The vibration amplitude of the rolling ball.

To prepare the Ti-6Al-4V alloy sample for USRP treatment, the turning process was performed with a cutting speed of 60 m/min, a feed rate of 0.05 mm/r, and a cut depth of 1 mm. Subsequently, the grinding process was performed with the wheel speed of 2000 r/min, workpiece rotation speed of 120 r/min, feed rate of 0.05 mm/r, and grinding depth of 0.05 mm. The dimension of the fatigue sample is shown in Figure 5. The USRP experimental setup and schematic of the USRP process are displayed in Figure 6. Table 3 presents the basic USRP parameters. The rolling ball was propelled by the ultrasonic vibration system, which was employed to strike the workpiece surface during USRP. A force sensor was mounted between the ultrasonic impact device and the spring device. The static force applied on the workpiece surface could be adjusted by controlling the compression length of the spring. The value of static force, which is measured by the force sensor, is displayed on the digital monitor. As shown in Figure 7, the ultrasonic amplitude of the rolling ball is 4 μm, which could be measured by the laser displacement sensor (NLV-250, Ploytec Measurement Technology Co., Waldbronn, Germany). To guarantee the dependability of experimental data, a minimum of three samples were processed in each situation.

### 2.4. Finite Element Simulation of USRP

**Figure 8:**
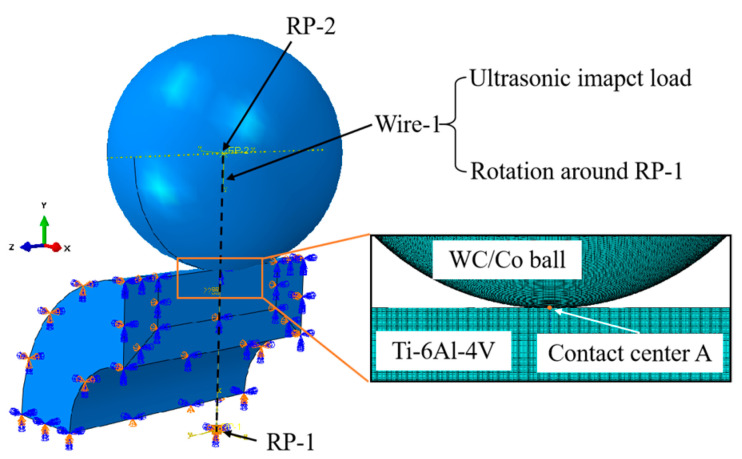
Finite element model of USRP.

The USRP was simulated by the ABAQUS/Explicit Finite element software (ABAQUS 2020), and the finite element model was established, as shown in Figure 8. The model of the workpiece was configured as a quarter of the hollow bar with an outer diameter of 20 mm and an inner diameter of 4 mm. The mesh size of the contact region between the rolling ball and the workpiece surface was refined to 50 μm × 50 μm for the purpose of minimizing computation time. The mechanical and thermal properties of the rolling ball are shown in Table 4. The elastic modulus of the rolling ball was much larger than the Ti-6Al-4V alloy, thus defining the rolling ball as an un-deformable rigid body. The thermo-physical properties of the rolling ball and the friction coefficient between the cemented carbide rolling ball and the Ti-6Al-4V workpiece could affect the temperature in the contact area during USRP. The friction coefficient between the WC/Co cemented carbide and Ti-6Al-4V was measured as 0.3 [32]. The other simulation settings were as follows: (1) Setting reference point 1 (RP-1) and reference point 2 (RP-2) at the center of the workpiece and the center of the rolling ball, respectively; (2) Creating a coupling constraint between the workpiece and RP-1; (3) Fixing the reference point 1 (RP-1) completely; (4) Setting a line constraint (Wire-1) between RP-1 and RP-2. An ultrasonic impact load was applied to Wire-1, which consisted of the static load and dynamic ultrasonic impact loads. The line constraint (Wire-1) rotated around RP-1. The simulation parameters were consistent with the parameters of the actual USRP test in Table 3; (5) Node A was placed in the center of the contact area, which was used to assess the evolution of the temperature and strain rate of the workpiece during USRP. Moreover, the J-C model was selected to describe the mechanical properties of Ti-6Al-4V, which was defined as follows:

(1) $$\sigma =(A+B\varepsilon^{n})(1+Cln\dot{\varepsilon }*)(1-{T*}^{m})$$

where A, B, C are the yield strength, the strain hardening modulus, and the strain rate hardening modulus, respectively. n is the hardening rate factor; ε is the plastic strain rate; ${\dot{\varepsilon }}^{*}$ is the strain rate factor, and m is the thermal influence factor. Table 5 summarizes the constitutive model parameters of Ti-6Al-4V.

### 2.5. Fatigue Test

**Figure 9:**
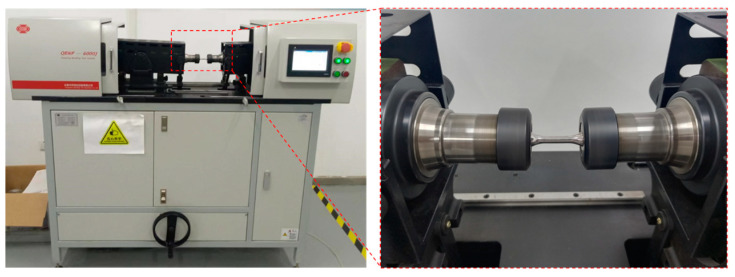
Rotational bending fatigue testing machine.

The rotational bending fatigue tests were conducted on the fatigue testing machine (QBWP-6000J, Changchun Qian Bang Test Equipment Co., Ltd., Changchun, China), as shown in Figure 9. The fatigue test was conducted with a maximum stress level of 800 MPa (80% of yield strength) at room temperature (25 °C), according to the general principle of fatigue test. Some other parameters of the fatigue test are shown in Table 6. To guarantee data reliability, a minimum of three fatigue test samples were employed in every machining condition.

### 2.6. Characterization Methodologies

#### 2.6.1. Surface Morphology and Roughness

A Zeiss LSM900 laser scanning confocal microscope (Zeiss, Jena, Germany) was utilized to perform the characterization of the 3D topography and surface roughness values of the machined Ti-6Al-4V workpiece. The surface roughness measurements for each sample were averaged from 10 different points on the workpiece to ensure experimental accuracy. The cross-sectional microstructure, machined surface morphology, and fatigue fracture morphology were characterized by utilizing a Phenom-XL scanning electron microscope (Phenom-World, Eindhoven, The Netherlands).

#### 2.6.2. Hardness Test

The cross-sectional hardness of the Ti-6Al-4V workpiece after USRP was tested utilizing a Vickers hardness tester (DUH-211, SHIMADZU, Kyoto, Japan). The hardness testing was performed by applying a maximum load of 1961 mN while employing a square-based diamond pyramid indenter. It was crucial to note that hardness tests were performed ten times on each workpiece to ensure an accurate analysis of the hardness.

#### 2.6.3. Residual Stress

The residual stress of the Ti-6Al-4V workpiece after USRP was measured by using the Stresstech DR45 residual stress testing equipment (Stresstech, Tikkutehtaantie, Jyväskylä, Finland). In order to assess the distribution of residual stress at different depths along the cross-sectional direction, the machined surface of the Ti-6Al-4V workpiece was treated by using the electrolytic polishing technique. This process employed the polishing solution, which was characterized by a combination of 10% HF, 27% HNO_2_, and 63% H_2_O. In addition, the {213} diffraction plane of hcp α-Ti with a Bragg angle of 143° was chosen, and the Cr-kα radiation source was selected during the measurement.

## 3. Results and Discussion

### 3.1. Dynamic Thermo-Mechanical Properties

**Figure 10:**
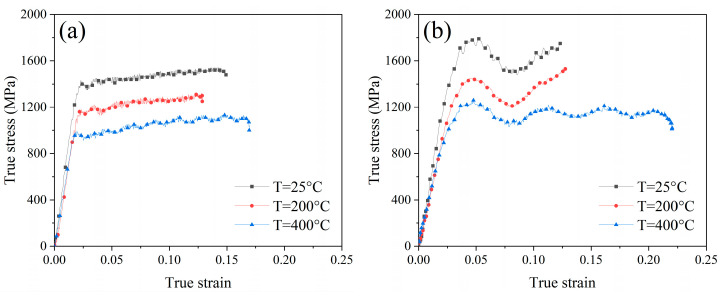
The true stress–strain curves of Ti-6Al-4V under various temperatures and strain rate: (**a**) strain rate of 2000 s^−1^; (**b**) strain rate of 4000 s^−1^.

The true stress–strain behavior of Ti-6Al-4V under the high strain rates and temperatures was measured, as shown in Figure 10. The yield strength and ultimate strength under different conditions were determined by the true stress–strain curve. Table 7 displays the dynamic thermo-mechanical properties of Ti-6Al-4V. Under the same temperature conditions, it could be observed that both yield strength and ultimate strength of Ti-6Al-4V material were enhanced when the strain rate of deformation raised from 2000 s^−1^ to 4000 s^−1^. This result demonstrated the existence of the strain rate strengthening effect. Conversely, under the same strain rate condition, it was found that both the yield strength and ultimate strength of Ti-6Al-4V material declined when the temperature increased from 25 °C to 400 °C, which performed as the thermal softening effect. In addition, the true stress–strain behavior of Ti-6Al-4V under the high strain rate and temperature could be determined by the combination of the strain hardening effect and thermal softening effect. The high strain rate plastic deformation of Ti-6Al-4V material could induce heat generation during the Split Hopkinson pressure bar (SHPB) experiment, which could lead to the thermal softening of the material. The strain hardening effect was stronger than the thermal softening effect during the deformation strain rate of 2000 s^−1^, so the true stress–strain curve measured was relatively smooth. However, the heat generation increased when the deformation strain rate reached 4000 s^−1^, and the true stress–strain curve oscillated could be due to the unsteady state between the strain hardening and thermal softening of the material. As for the USRP treatment, it was characterized as a thermo-mechanical coupling process due to the friction and impact effect between the rolling ball and the workpiece. Therefore, the dynamic thermo-mechanical properties of Ti-6Al-4V could be associated with the surface deformation behavior under the USRP of the Ti-6Al-4V workpiece.

### 3.2. Numerical Simulation Results

**Figure 11:**
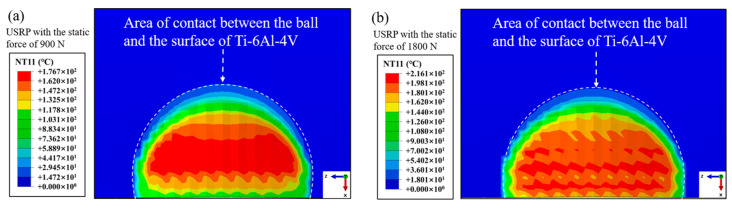
The surface temperature field of workpiece under USRP treatment with different static forces: (**a**) static force of 900 N; (**b**) static force of 1800 N.

**Figure 12:**
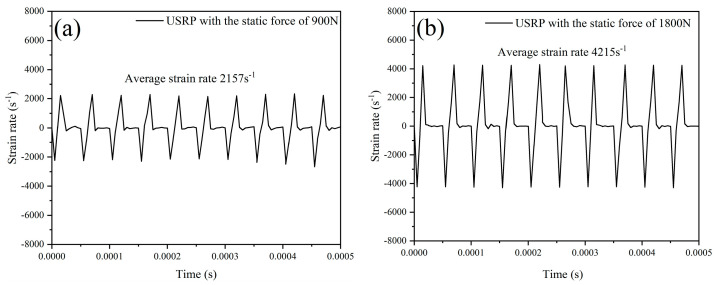
The evolution of strain rate in the center of contact region under USRP treatment with different static forces: (**a**) static force of 900 N; (**b**) static force of 1800 N.

Figure 11 displays the surface temperature field in the contact region between the rolling ball and Ti-6Al-4V workpiece under USRP with various static forces. It was found that the temperature in the center of the contact region could reach 176 °C and 216 °C when the USRP parameter of static force was 900 N and 1800 N, respectively. This could be due to the frictional heating and plastic deformation work between the rolling ball, and the workpiece was converted to thermal energy, which led to the increase in temperature in the contact area. The strain rate of deformation in the center of the contact region between the rolling ball and Ti-6Al-4V workpiece under USRP treatment with various static forces is shown in Figure 12. It was observed that the strain rate of deformation in the center of the contact region could reach 2157 s^−1^ and 4215 s^−1^ when the USRP parameter of static force was 900 N and 1800 N, respectively. The coupling effect of the static force and dynamic ultrasonic impact force during USRP treatment could result in high instantaneous velocity and acceleration at the tip of the rolling ball, which could lead to severe plastic deformation of the workpiece.

**Figure 13:**
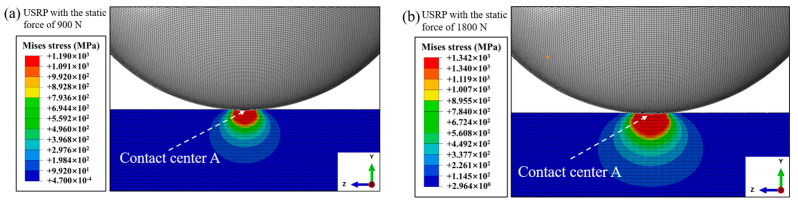
The Mises stress under USRP treatment at different static forces: (**a**) static force of 900 N; (**b**) static force of 1800 N.

**Figure 14:**
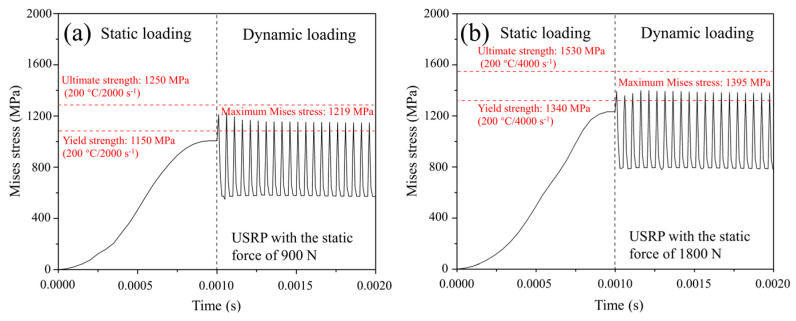
Stress–time curves at contact center between rolling ball and Ti-6Al-4V specimen for different USRP parameters: (**a**) static force of 900 N; (**b**) static force of 1800 N.

Figure 13 illustrates the distribution of Mises stress when the rolling ball is pressed into the deepest position of the Ti-6Al-4V workpiece surface during USRP treatment. In general, plastic deformation could be generated under the condition of the suffered Mises stress being larger than the yield strength of the material. It could be observed that the maximum Mises stress appeared on the workpiece surface, and it decreased gradually along the in-depth direction. Additionally, the distribution area of Mises stress was expanded as the USRP parameter of static force increased. Figure 14 shows the stress–time curve of the center of contact region (point A) between the rolling ball and Ti-6Al-4V workpiece during USRP treatment. The Mises stress–time curve was divided into the static loading stage and the ultrasonic cyclic impact loading stage. In the dynamic cyclic impact stage, it could be calculated that the maximum Mises stress in the center of the contact region could reach 1219 MPa and 1395 MPa when the USRP parameter of static force was 900 N and 1800 N, respectively. According to the yield strength and ultimate strength, which were measured from the dynamic true stress–strain curves of Ti-6Al-4V, it showed that the yield strength and ultimate strength were 1150 MPa and 1250 MPa under the test conditions with a temperature of 200 °C and strain rate of 2000 s^−1^. Similarly, the yield strength and ultimate strength were 1340 MPa and 1530 MPa under the test conditions with temperature of 200 °C and strain rate of 4000 s^−1^. It could be noted that the maximum Mises stress was between the yield strength value and ultimate strength value under the corresponding conditions. Therefore, it could be claimed that the plastic deformation occurred at the contact region between the rolling ball and Ti-6Al-4V workpiece, which belonged to the mechanism of strain strengthening during USRP treatment.

### 3.3. Analysis of Surface Morphology and Roughness

**Figure 15:**
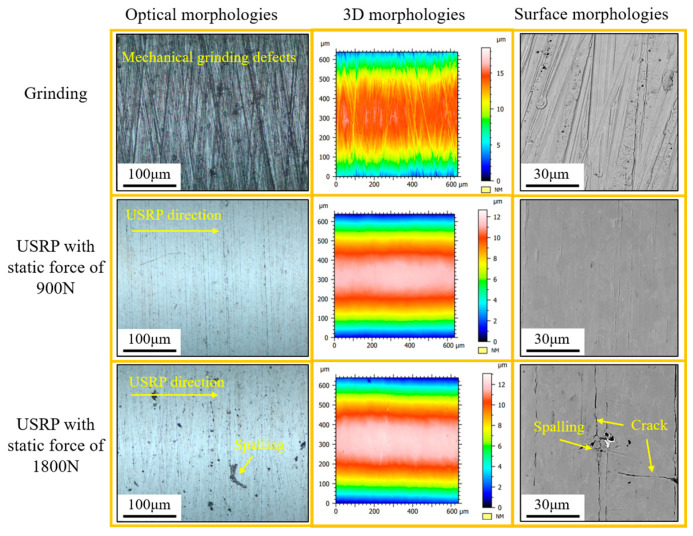
Surface morphologies of Ti-6Al-4V workpiece after grinding and USRP treatment.

The surface morphologies of the Ti-6Al-4V workpiece after grinding and USRP treatment were measured, as shown in Figure 15. It showed that the surface of the Ti-6Al-4V workpiece after grinding exhibited irregular machining defects, which were characterized as sharp grooves caused by the sliding effect of abrasive. The bottom of these sharp grooves was highly susceptible to inducing the stress concentration, which promoted the initiation and propagation of fatigue cracks during the service condition of the workpiece. However, as for the USRP treatment of the Ti-6Al-4V workpiece, the plastic flow of material could occur on the surface, causing the sharp grooves to fill and the surface roughness to decrease. Therefore, it could be observed that the surface morphology of the Ti-6Al-4V workpiece exhibited smoother under the USRP treatment with the static force of 900 N. With an increase in static force to 1800 N during USRP, micro-defects, such as cracking and spalling, appeared on the machined surface of the workpiece. It could be inferred that the contact effect between the rolling ball and workpiece during USRP included normal pressure, rolling friction, and sliding friction. The severe plastic deformation and greater sliding friction effect were generated when the excessive static force was applied to the workpiece surface during USRP. This could induce crack initiation and surface damage with the influence of tensile stress. Thus, the surface quality of the Ti-6Al-4V workpiece may be seriously affected by the unsuitable process parameters of the USRP treatment.

**Figure 16:**
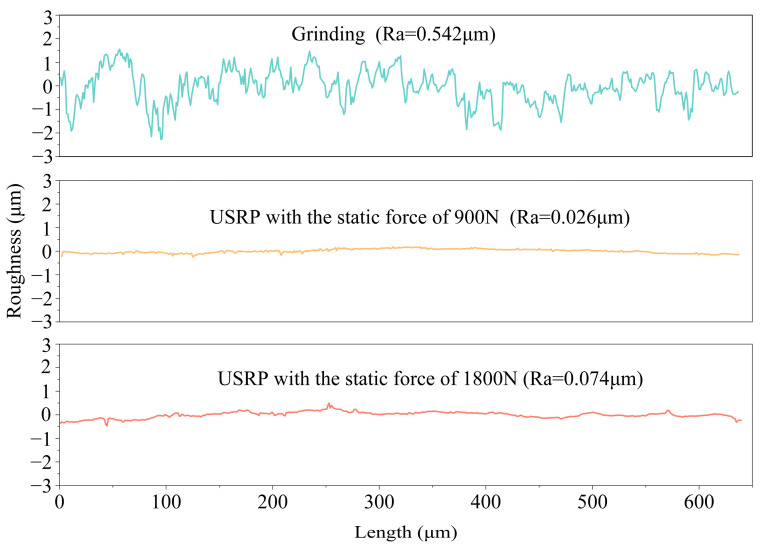
Surface roughness value of Ti-6Al-4V workpiece after grinding and USRP treatment.

Figure 16 presents the comparison of the surface roughness value of the Ti-6Al-4V workpiece after grinding and USRP treatment. The surface roughness value of the Ti-6Al-4V workpiece after grinding was 0.542 μm, which was effectively reduced to 0.026 μm after USRP reached the static force of 900 N. However, the surface roughness value of the workpiece increased to 0.074 μm after USRP reached the static force of 1800 N. The decline in surface roughness of the Ti-6Al-4V workpiece by using USRP treatment could contribute to preventing the occurrence of fatigue cracks, thereby enhancing the fatigue resistance of the workpiece.

### 3.4. Microstructure Analysis

**Figure 17:**
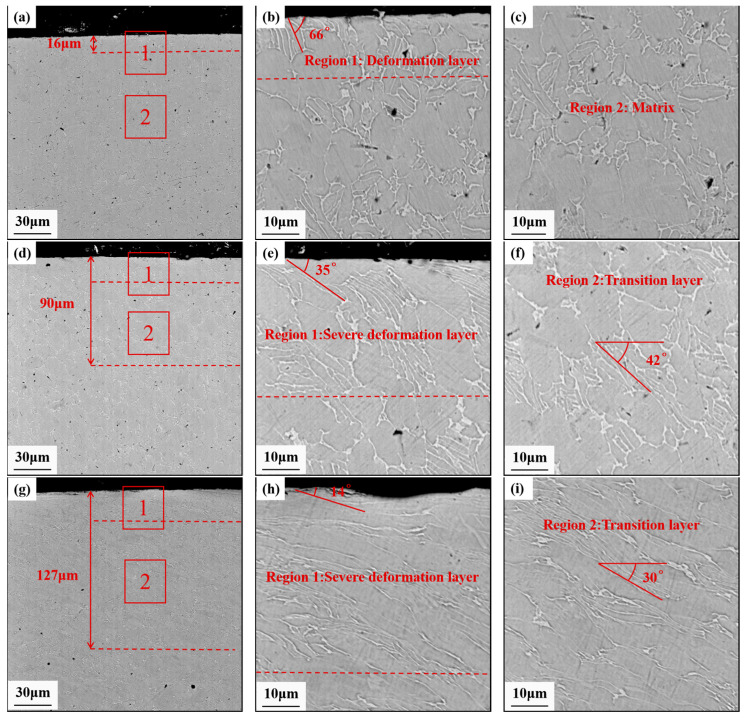
Cross-sectional SEM morphologies of Ti-6Al-4V workpieces: (**a**) Grinding; (**b**) Enlarged view of region 1 in (**a**); (**c**) Enlarged view of region 2 in (**a**); (**d**) USRP with the static force of 900 N; (**e**) Enlarged view of region 1 in (**d**); (**f**) Enlarged view of region 2 in (**d**); (**g**) USRP with the static force of 1800 N; (**h**) Enlarged view of region 1 in (**g**); (**i**) Enlarged view of region 2 in (**g**).

Figure 17 shows the evolution of the cross-sectional microstructure of the Ti-6Al-4V workpiece after grinding and USRP treatment. The deformation layer of the Ti-6Al-4V workpiece after grinding was approximately 16 μm. The transformed β phases were slightly elongated and inclined with an angle of 66° along the surface. The plastic deformation depth increased rapidly after USRP treatment, which could be divided into a severe deformation layer and a transition layer. The surface of the workpiece was subjected to the combination of the dynamic ultrasonic impact force and shear force during USRP. In the severe deformation layer, the transformed β phases were significantly bent and elongated along the rolling direction. The transformed β phases gradually became parallel to the surface of the workpiece with the increase in the static force. When the static force of USRP increased from 900 N to 1800 N, the depth of the plastic deformation layer expanded from 90 μm to 127 μm, and the inclined deformation angle decreased from 35° to 14°. The transition layer was situated between the severe deformation layer and the matrix, which was further away from the surface. The applied load gradually decreased in this region, which resulted in a reduction in the bending deformation of the transformed β phases. The static force applied to the rolling ball could make it contact tightly with the Ti-6Al-4V workpiece during USRP. It could contribute to the high-frequency ultrasonic impact energy continuously transferred to the workpiece surface. With an increase in static force, the energy transfer between the rolling ball and the workpiece surface could be higher, leading to a larger plastic deformation region. Simultaneously, under the thermo-mechanical coupling condition of USRP treatment, the severe extrusion force could result in grain refinement, causing the coarse crystalline structure to bend and deform in a specific direction [33].

### 3.5. Hardness Analysis

**Figure 18:**
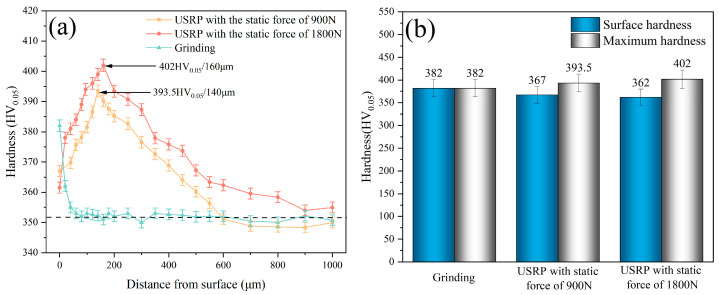
Hardness distribution of Ti-6Al-4V workpiece after grinding and USRP treatment: (**a**) Hardness distribution tendency in the cross-section of workpiece; (**b**) Surface hardness and maximum hardness of the workpiece.

Figure 18 illustrates the hardness distribution of the Ti-6Al-4V workpiece after grinding and USRP treatment. The hardness of the Ti-6Al-4V matrix was 353 HV_0.05_. The surface of the original workpiece suffered plastic deformation and hardening after grinding. This led to an increase in the maximum hardness to 382 HV_0.05_, and the maximum hardness value appeared at the workpiece surface. The trend of hardness value from the machined surface to the subsurface layer after grinding decreased. The depth of the work hardening layer was approximately 40 μm. Figure 18a illustrates the hardness distribution from the machined surface to the subsurface layer after USRP with different static forces. The maximum hardness could reach 393.5 HV_0.05_ after USRP treatment with the static force of 900 N, which was 11.47% higher than the matrix hardness. The depth of maximum hardness value was about 140 μm. The maximum hardness could reach 402 HV_0.05_ after USRP treatment with the static force of 1800 N, which was 13.88% higher than the matrix hardness. The depth of maximum hardness value was about 160 μm. This was attributed to the increase in the static force, which led to a greater level of plastic deformation behavior on the workpiece surface. The trend of hardness distribution in the cross-section of the workpiece after USRP was shown to increase at first and then decrease to the matrix hardness. In addition, according to the Hertz contact principle, the maximum strain in the subsurface layer of the workpiece after USRP could occur below the surface [34]. This could be associated with the maximum hardness value appearing at the subsurface of the workpiece after the USRP treatment.

### 3.6. Residual Stress Analysis

**Figure 19:**
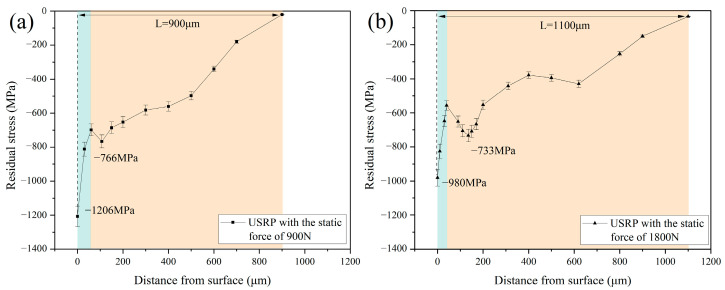
The distribution of residual stress after USRP with different static forces: (**a**) static force of 900 N; (**b**) static force of 1800 N.

The plastic deformation of the Ti-6Al-4V workpiece during USRP induced the formation of the residual compressive stress layer and grain refinement. This layer could contribute to preventing the propagation of fatigue cracks, leading to an enhancement of the fatigue resistance properties of the workpiece. Figure 19 shows the residual stress distribution of the Ti-6Al-4V workpiece after USRP treatment. The residual compressive stress of the machined Ti-6Al-4V surface after USRP with the static force of 900 N could reach 1206 MPa. However, when the static force of USRP further increased to 1800 N, the residual compressive stress of the machined Ti-6Al-4V surface decreased to 980 MPa. As mentioned in Section 3.3, the excessive increase in static force during USRP could lead to an aggravation of the surface quality of the workpiece. The presence of cracking and spalling on the workpiece surface could induce the attenuation of the residual compressive stress. In addition, it was observed that the residual compressive stress value at the topmost surface of the Ti-6Al-4V workpiece was the maximum. This could be associated with the formation of a thin layer on the topmost surface of the workpiece after USRP, which could be characterized as the combination of nanograins and amorphous material [35]. The distribution of residual compressive stress in the workpiece subsurface layer after USRP showed a decrease first and an increase later, finally decreasing to zero. As the static force of USRP increased from 900 N to 1800 N, it could be noted that the depth of the residual compressive stress layer of the workpiece improved from 900 μm to 1100 μm.

### 3.7. Fatigue Properties Analysis

**Figure 20:**
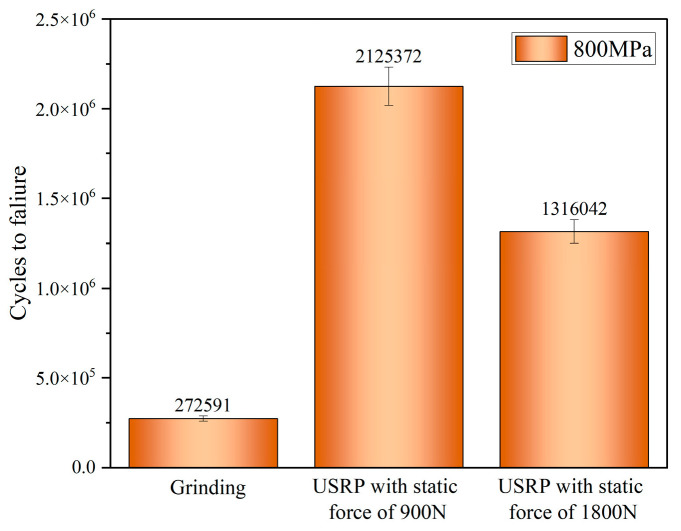
Fatigue life of Ti-6Al-4V workpiece after grinding and USRP treatment at fatigue stress of 800 MPa during the rotational bending fatigue tests.

The fatigue life of the Ti-6Al-4V workpiece after grinding and USRP treatment was evaluated at fatigue stress of 800 MPa during the rotational bending fatigue tests, as shown in Figure 20. As for the Ti-6Al-4V workpiece, after grinding, the fatigue life reached about 272,591 cycles at a stress level of 800 MPa. After USRP treatment with the static force of 900 N, the fatigue life reached about 2,125,372 cycles at fatigue stress of 800 MPa, which improved by about 7.8 times in fatigue performance compared with the grinding workpiece. After USRP treatment with the static force of 1800 N, the fatigue life reached about 1,316,042 cycles at fatigue stress of 800 MPa, which improved by about 4.82 times in fatigue performance compared to the grinding workpiece. It could be observed that the fatigue life of the Ti-6Al-4V workpiece after USRP treatment with the static force of 1800 N was lower than that of USRP with the static force of 900 N. This could be due to the formation of better surface integrity and greater residual compressive stress after USRP treatment with the static force of 900 N. Nonetheless, it could be concluded that the fatigue life of the Ti-6Al-4V workpiece after USRP treatment was greatly improved compared with the fatigue life of the workpiece after grinding.

**Figure 21:**
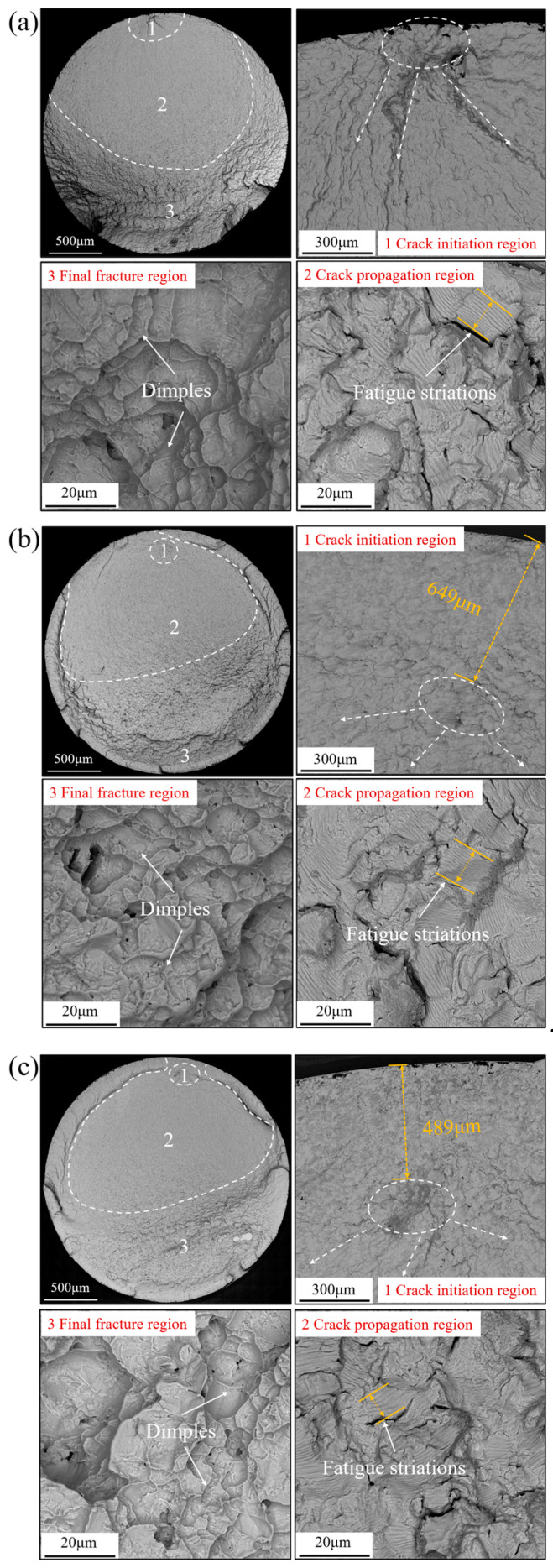
Fatigue fracture morphology of Ti-6Al-4V workpiece with different processing methods under the fatigue stress of 800 MPa: (**a**) Grinding; (**b**) USRP with static force of 900 N; (**c**) USRP with static force of 1800 N.

The fracture morphologies of the Ti-6Al-4V workpiece after fatigue tests with different processing methods under the fatigue stress of 800 MPa are shown in Figure 21. The fatigue fracture image could be classified into three distinct regions: crack initiation region (Region 1); crack propagation region (Region 2); and final fracture region (Region 3). Figure 21a shows the fatigue fracture diagram of the workpiece with grinding treatment. It could be observed that the fatigue crack source area appeared at the workpiece surface. Microcracks appeared near the fatigue source in the crack initiation region (Region 1), which could be attributed to the crack propagation region (Region 2). It could be noted that the dimples were captured in the final fracture region (Region 3), which indicated that the ductile fracture behavior occurred. Figure 21b shows the fatigue fracture diagram of the workpiece after USRP with a static force of 900 N. It could be observed that the fatigue crack source area appeared at a distance of about 649 μm below the workpiece surface. This could be associated with the formation of the great residual compressive stress field in the workpiece subsurface after USRP, which could contribute to the inhibition of the crack propagation to the workpiece surface. Similarly, the microcracks and dimples could be found in the crack propagation region and the final fracture region, respectively. Figure 21c shows the fatigue fracture diagram of the workpiece after USRP with a static force of 1800 N. The fatigue crack source area appeared at a distance of about 489 μm below the workpiece surface. By increasing the static force of USRP from 900 N to 1800 N, it was observed that the distance between the fatigue fracture source and the workpiece surface decreased significantly, which could have a negative influence on the fatigue resistance properties of the workpiece. The fatigue crack was initiated at the defective locations and propagated by the tensile stress. The formation of a greater residual compressive stress field after USRP with the static force of 900 N could promote the fatigue crack source being transferred to the deeper position of the workpiece. In contrast, the excessive static force of 1800 N applied on the workpiece could induce damage failure and eliminate the beneficial effect of USRP treatment. This indicates that the selection of the appropriate processing parameters for USRP treatment is very critical.

## 4. Conclusions

This study investigated the surface integrity, microstructural evolution, hardness distribution, and residual stress field of the Ti-6Al-4V workpiece after the ultrasonic surface rolling process (USRP) with different static forces. The fatigue resistance properties of the workpiece after USRP treatment were further analyzed. The deformation strain rate and contact temperature between the rolling ball and Ti-6Al-4V during USRP were analyzed by numerical simulation. Additionally, the correlation between the true thermo-mechanical behavior of Ti-6Al-4V material and the USRP parameters of the Ti-6Al-4V workpiece was established. The conclusions are as follows:

(1) The strain rate of deformation in the center of the contact region could reach 2157 s^−1^ and 4215 s^−1^ when the USRP parameter of static force was 900 N and 1800 N, respectively. The coupling effect of the static force and dynamic ultrasonic impact force during USRP treatment could result in the high instantaneous velocity and acceleration at the tip of the rolling ball, which could lead to the severe plastic deformation of the workpiece;
(2) The surface roughness value of the Ti-6Al-4V workpiece after grinding was 0.542 μm, which was effectively reduced to 0.026 μm after USRP with the static force of 900 N. However, the surface roughness value of the workpiece increased to 0.074 μm after USRP with the static force of 1800 N. The decline in surface roughness of the Ti-6Al-4V workpiece by using USRP treatment could contribute to preventing the occurrence of fatigue cracks, thereby enhancing the fatigue resistance of the workpiece;
(3) The static force applied to the rolling ball could make it contact tightly with the Ti-6Al-4V workpiece during USRP. It could contribute to the high-frequency ultrasonic impact energy continuously transferred to the workpiece surface. With an increase in static force, the energy transfer between the rolling ball and the workpiece surface could be higher, leading to a larger plastic deformation region. Simultaneously, under the thermo-mechanical coupling condition of USRP treatment, the severe extrusion force could result in grain refinement, causing the coarse crystalline structure to bend and deform in a specific direction;
(4) The trend of hardness distribution in the cross-section of the workpiece after USRP was shown to increase at first and then decrease to the matrix hardness. The residual compressive stress value at the topmost surface of the Ti-6Al-4V workpiece was the maximum. This could be associated with the formation of a thin layer on the topmost surface of the workpiece after USRP, which could be characterized as the combination of nanograins and amorphous material. The distribution of residual compressive stress in the workpiece subsurface layer after USRP showed as decreasing early and increasing later, finally decreasing to zero;
(5) The fatigue life of the Ti-6Al-4V workpiece after USRP treatment with the static force of 1800 N was lower than that of USRP with the static force of 900 N. This could be due to the formation of better surface integrity and greater residual compressive stress after USRP treatment with the static force of 900 N. Nonetheless, the fatigue life of the Ti-6Al-4V workpiece after USRP treatment greatly improved compared with the fatigue life of the workpiece after grinding. Microcracks appeared near the fatigue source in the crack initiation region. The dimples were captured in the final fracture region, which indicated that the ductile fracture behavior occurred. The excessive static force applied on the workpiece could induce damage failure and eliminate the beneficial effect of USRP treatment.

## Figures and Tables

**Table 1:** Chemical composition of Ti-6Al-4V (wt%).

Al	V	O	Fe	C	N	H	Ti
6.19	4.08	0.13	0.029	0.012	0.012	0.001	Balance

**Table 2:** Dynamic mechanical properties test parameters.

Strain Rate (s^−1^)	Temperature (°C)
2000	25/200/400
4000	25/200/400

**Table 3:** The basic USRP parameters.

Static Force (N)	Rotating Speed (r/min)	Feed Rate (mm/r)	Ultrasonic Frequency (kHz)	Ultrasonic Amplitude (μm)	Processing Pass
900/1800	100	0.05	27	4	1

**Table 4:** The mechanical and thermal properties of the rolling ball.

Materials	λ/Thermal Conductivity (W·m^−1^·K^−1^)	ρ/Density (kg/m^3^)	Poisson’s Ratio	E/Elastic Modulus (GPa)	α/Linear Expansion Coefficient (10^−6^)	γ/Specific Heat J/(kg·K)
WC/Co cemented carbide	50	12,500	0.23	530	6.2	165

**Table 5:** The constitutive model parameters of Ti-6Al-4V.

A/Yield Strength (GPa)	B/Strain Hardening (GPa)	n/Hardening Rate Factor	m/Thermal Influence Factor	Melting Temperature (K)	Reference Temperature (K)	C/Empirical Strain Rate Sensitivity Coefficient	$\dot{\varepsilon }*$/Strain Influence Factor
1.05	2.63	0.6656	1.2949	1848	298	0.0825	1

**Table 6:** Parameters of fatigue test.

Frequency (Hz)	Stress Ratio	Fatigue Stress Level (MPa)	Test Temperature (°C)
50	−1	800	25

**Table 7:** Dynamic thermo-mechanical properties of Ti-6Al-4V.

Strain Rate (s^−1^)	Temperature (°C)	Yield Strength (MPa)	Ultimate Strength (MPa)
2000	25	1400	1480
	200	1150	1250
	400	955	1070
4000	25	1680	1760
	200	1340	1530
	400	1150	1210

## Data Availability

The data that support the findings of this study are available on request from the corresponding author.
